# Performance characteristics of finger-stick dried blood spots (DBS) on the determination of human immunodeficiency virus (HIV) treatment failure in a pediatric population in Mozambique

**DOI:** 10.1371/journal.pone.0181054

**Published:** 2017-07-13

**Authors:** Joy Chang, Amina de Sousa, Jennifer Sabatier, Mariamo Assane, Guoqing Zhang, Dulce Bila, Paula Vaz, Charity Alfredo, Loide Cossa, Nilesh Bhatt, Emilia H. Koumans, Chunfu Yang, Emilia Rivadeneira, Ilesh Jani, James C. Houston

**Affiliations:** 1 Centers for Disease Control and Prevention (CDC), Atlanta, GA, United States of America; 2 Instituto Nacional de Saúde (INS), Ministry of Health, Maputo, Mozambique; 3 Fundação Ariel Glaser contra o SIDA Pediátrico (Ariel), Maputo, Mozambique; 4 Centers for Disease Control and Prevention, Maputo, Mozambique; Universita degli Studi di Roma Tor Vergata, ITALY

## Abstract

Quantitative plasma viral load (VL) at 1000 copies /mL was recommended as the threshold to confirm antiretroviral therapy (ART) failure by the World Health Organization (WHO). Because of ongoing challenges of using plasma for VL testing in resource-limited settings (RLS), especially for children, this study collected 717 DBS and paired plasma samples from children receiving ART ≥1 year in Mozambique and compared the performance of DBS using Abbott’s VL test with a paired plasma sample using Roche’s VL test. At a cut-off of 1000 copies/mL, sensitivity of DBS using Abbott DBS VL test was 79.9%, better than 71.0% and 63.9% at 3000 and 5000 copies/mL, respectively. Specificities were 97.6%, 98.8%, 99.3% at 1000, 3000, and 5000 copies/mL, respectively. The Kappa value at 1000 copies/mL, 0.80 (95% CI: 0.73, 0.87), was higher than 0.73 (95% CI: 0.66, 0.80) and 0.66 (95% CI: 0.59, 0.73) at 3000, 5000 copies/mL, respectively, also indicating better agreement. The mean difference between the DBS and plasma VL tests with 95% limits of agreement by Bland-Altman was 0.311 (-0.908, 1.530). Among 73 children with plasma VL between 1000 to 5000 copies/mL, the DBS results were undetectable in 53 at the 1000 copies/mL threshold. While one DBS sample in the Abbott DBS VL test may be an alternative method to confirm ART failure at 1000 copies/mL threshold when a plasma sample is not an option for treatment monitoring, because of sensitivity concerns between 1,000 and 5,000 copies/ml, two DBS samples may be preferred accompanied by careful patient monitoring and repeat testing.

## Introduction

In 2013, WHO recommended monitoring quantitative viral load (VL) as the preferred approach to diagnose antiretroviral treatment (ART) failure; plasma HIV viral load of 1000 copies/ml was recommended as the threshold [1, 2]. However, in resource-limited settings (RLS), using plasma for VL testing presents substantial challenges because of the stringent collection and processing requirements including whole blood collection by venipuncture, plasma separation, cold chain transportation within limited hours, and appropriate short and long-term plasma storage. Using plasma for VL testing is especially challenging for small children since venipuncture is more difficult and experienced phlebotomists are often not available.

In order to provide universal VL monitoring in RLS, an alternative sample for VL testing with adequate performance characteristics at the recommended threshold, especially for children, is urgently needed. A dried blood spot (DBS) collected with a finger stick (FS-DBS) for early infant diagnosis of HIV (EID) is routine in many RLS, can be used for drug resistance (HIVDR) genotyping, and has a simple collection method and stability at room/ambient temperature [3–11]. Various studies have demonstrated the feasibility and performance of using DBS for viral load (VL) monitoring using Abbott’s VL test [12–19]. However, many of these studies had an insufficient sample size to generate robust estimates of performance characteristics such as sensitivity and specificity at different VL thresholds; the exception was presented by Schmitz at al. [20]. In Schmitz’s study, paired plasma and two DBS samples from 416 adults and 377 children on ART ≥6 months were compared. Results suggested that the two DBS samples provided a practical alternative to plasma in RLS for VL testing and the 1,000 copies/ml cut-off using DBS for VL was optimal for ARV treatment failure determination. In our study, we focused exclusively on children receiving ART to generate robust performance estimates of DBS VL to diagnose ART failure. We collected samples from over 700 children in Mozambique on ART for more than one year and compared the performance of one DBS VL sample with paired plasma VL using 1000 (the WHO standard), 3000, and 5000 copies as thresholds.

## Methods and materials

### Study setting

We conducted a cross-sectional study in urban clinics providing pediatric HIV care in Maputo province from August 2013—March 2014. Six public health clinics meeting these criteria were selected: 1) >400 children enrolled on ART; 2) prior experience in collecting DBS specimens for EID; and 3) located within 30 minutes’ drive of the Instituto Nacional de Saude (INS). Overall 16% of children on ART in Mozambique were included in this evaluation.

### Study population

Children who were aged 1 to 15 years old and had been on ART for longer than 12 months at the time of recruitment were eligible for inclusion. From August 2013 to March 2014, a total of 723 children were consecutively enrolled. The pediatric ART regimens used were zidovudine, lamivudine and nivarapine for children older than 2 years old and stavudine, lamivudine and nevirapine for children less than 2 years old. Lopinavir/ritonavir replaced nevirapine in children who had history of exposure to nevirapine as part of prevention of mother-to-child transmission (PMTCT).

### Blood collection and sample preparation

Among the 723 children enrolled, 717 (99.2%) paired plasma and FS-DBS samples were successfully collected. From each patient, 5 mL of venous whole blood was collected into an ethylenediaminetetraacetic acid (EDTA) anticoagulant tube and transported cold within 6 hours of sample collection to INS in Maputo for plasma separation [21, 22], and ~0.5 mL from a finger stick was collected into a Micro-EDTA tube (BD Diagnostics, New Jersey, USA) for DBS preparation. Whole blood from FS EDTA was dotted onto Whatman 903 filter paper using a plastic transfer pipette. Each blood spot contained 70 to 75 μl of EDTA-whole blood; a total of five DBS were collected for each patient. The DBS cards were dried at least 6 hours at room temperature. Once dried, the DBS samples were separated with glassine paper, packed in low-gas–permeable plastic bags with desiccants, and transported to the INS at ambient temperature. At the INS, the DBS samples were stored at –80°C and shipped with dried ice in batches to the Centers for Disease Control and Prevention (CDC), Atlanta, Georgia, USA, for testing. The DBS packaging and shipping followed international shipping guidelines and regulations [23].

### HIV-1 viral load (VL) quantification using plasma samples

The plasma HIV-1 VL was measured using Roche COBAS CAP/CTM HIV-1 Test, version 2.0 (Roche Diagnostics, Johannesburg, South Africa) at the INS following the manufacturer’s instructions [21]. Briefly, 1.1 ml of plasma from each patient was processed for the HIV-1 RNA isolation and VL quantification on the COBAS Amplipre/COBAS TaqMan with the Amplilink software 3.3.

### HIV-1 viral load quantification using DBS samples

DBS VL quantification performed at the CDC, Atlanta, USA, using the Abbott RealTime HIV-1 Assay (Abbott Laboratories, Chicago, IL, USA) [24] was performed blindly without any knowledge of any treatment or previous testing results. Briefly, one blood spot per patient was removed from the DBS card with sterile scissors, placed into a tube with 1.7 ml of mLysis buffer provided with the Abbott sample preparation system, and incubated at room temperature for 1 h with intermittent mixing. The RNA was extracted from the lysate, and VL was measured from the extracted RNA using Abbott RealTime HIV-1 test with m2000 DBS HIV-1 RNA ‘open-mode’ protocol (Abbott Molecular, Germany).

### Statistical analysis

All VL results were transformed to log_10_ values for analysis. Sensitivities and specificities of DBS at thresholds of 1000, 3000, and 5000 copies/mL were calculated using the paired plasma result as the reference standard. Cohen’s kappa was used to assess the agreement between the plasma and DBS VL tests. A kappa value of 0.80 indicates good concordance [25]. Bland-Altman was performed to show the correlation between the plasma and DBS VL testing results. The method employed for svy tabulate in Stata 13 was used to calculate 95% confidence intervals (CIs) for the misclassification of DBS for VL. The survey procedures were used to account for site-level correlations of data clustering within clinics. All statistical analyses were performed by using SAS software, version 9.3 (SAS Institute, Inc., Cary, North Carolina).

### Ethics review

The study was reviewed and approved by the Mozambican National Ethics Committee, Comité Nacional de Bioética para Saúde de Moçambique (IRB00002657) and Centers for Disease Control and Prevention (CDC) Human Research Protection Office (#5448). Written consent was obtained from a parent or legal guardian of the eligible child before enrollment.

## Results

Table 1 summarizes the demographic and clinical information of the 723 enrolled children; 717 (99.2%) provided paired plasma and FS-DBS samples and 713 had complete demographic information. The mean age at enrollment was 8 years and 6 months and 45.9% of these children were female (Table 1).

**Table 1:** Demographic* and clinical characteristics of children on ART in Mozambique at enrollment into DBS-plasma VL comparison study with 95% confidence interval (CI).

Age, sex, and ART (n = 713*)	**# of Children**	**Months**	**95% CI**
**Current Age (mean)**	713	102.9	[81.0, 124.7]
**Age at initiation of ART (mean)**	713	35.3	[24.9, 45.7]
**Time on ART (mean)**	713	60.3	[38.8, 81.2]
**Female (N, %)**	327 (45.9)		
Immunosuppression** (n = 591)	**# of Children**	**%**	**95% CI**
**None**	507	85.8	[75.2,92.3]
**Mild**	41	6.9	[4.6,10.3]
**Advanced**	18	3	[1.0,8.8]
**Severe**	25	4.2	[1.8,9.6]
Immunosuppression** at initiation **of ART (n = 616)**	**# of Children**	**%**	**95% CI**
**None**	98	15.9	[12.9,19.4]
**Mild**	62	10.1	[6.3,15.8]
**Advanced**	113	18.3	[14.8,22.5]
**Severe**	343	55.7	[47.7,63.4]
**WHO Stage (n = 682)**	** # of Children**	** %**	
**I**	66	9.3	[1.8,36.6]
**II**	156	22	[9.0,44.4]
**III**	340	47.9	[33.2,62.9]
**IV**	120	16.9	[7.2,34.8]
**WHO stage at ART Initiation (n = 625)**	**# of Children**	**%**	**95% CI**
**I**	75	10.5	[3.1,29.9]
**II**	165	23.1	[11.7,40.5]
**III**	307	43.1	[21.7,67.3]
**IV**	78	10.9	[5.7,20.0]

*Among the 723 children enrolled in the study, 713 children provided demographic information.

**CD4 levels in relation to the severity of Immunosuppression. None, > 500/mm^3^; Mild, 350–499/mm^3^; Advanced, 200-349/mm^3^; Severe, <200/mm^3^

**Fig 1:**
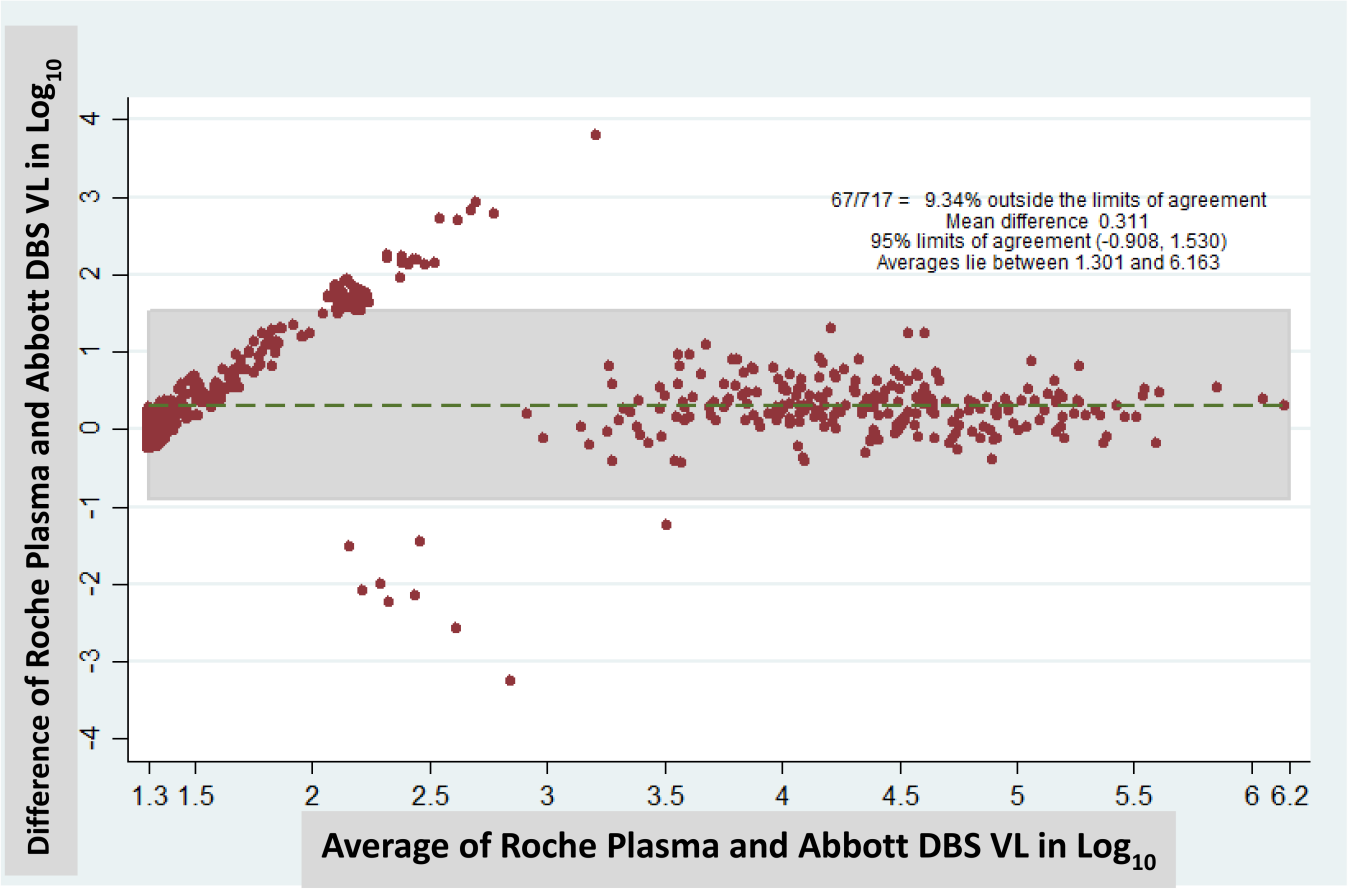
Bland-Altman analysis of Roche plasma VL and Abbott DBS VL among children on ART in Mozambique.

Using the plasma VL results as the reference standard, the DBS VL sample showed the highest sensitivity, 79.9% (60.9, 91.0) and the lowest false-negative rate, 20.1% (9.0, 39.1), when 1000 copies/mL was set as the threshold. The highest specificity, 99.5% (98.1, 99.9) and the lowest false- positive rate, 0.5% (0.1, 1.9), was observed when 5000 copies/mL was set as the threshold (Table 2). The Kappa value for the 1000 copies/mL DBS threshold was 0.80 (95% CI; 0.7, 0.9), higher than those for the 3000 and 5000 copies/mL. The Bland-Altman analyses in Fig 1 showed the mean difference between the Roche plasma and Abbott FS-DBS VL results was 0.311 and the 95% limits of agreement were between -0.908, and 1.530. Among 717 samples, 67 (9.3%) were outside of limits of agreement.

**Table 2:** Sensitivity, specificity, Kappa agreement, and false negativity and positivity of one Abbott DBS VL sample compared with the paired Roche plasma VL among children on ART in Mozambique.

					FALSE	FALSE
Plasma vs DBS		Sensitivity	Specificity	Kappa	negative	positive
(copies/mL)	N =	% (95% CI)	% (95% CI)	(95% CI)	% (95% CI)	% (95% CI)
1000:1000	717	79.9 (60.9, 91.0)	97.8 (95.0, 99.0)	0.80 (0.73, 0.87)	20.1 (9.0, 39.1)	2.2 (1.0, 5.0)
1000:3000	717	71.0 (51.8, 84.8)	99.0 (95.7, 99.8)	0.73(0.66, 0.80)	29.0 (15.2, 48.2)	1.0 (0.2, 4.3)
1000:5000	717	63.9 (47.7, 77.1)	99.5 (98.1, 99.9)	0.66 (0.59, 0.73)	36.4 (22.9, 52.3)	0.5 (0.1, 1.9)

N—Total number of samples with paired plasma and FS-DBS

When using the WHO-recommended threshold, 297 of 717 children (41.6%) had a plasma VL level greater than 1000 copies/mL, indicating a substantial rate of ART failure (Table 3). Of the 420 out of 717 samples with a plasma VL <1000 copies/mL (Table 3), 9 (2.1%) were classified as >1000 copies/mL by the DBS VL sample. For the 297 samples with plasma VL ≥1000 copies/mL, 72 (24.2%) were between 1000 and 5000 copies/mL, among which 20 (27.8%) were detected by the DBS sample and 52 were not. Among these 52 samples, 38 (73.1%) had plasma VL at 1000 copies/mL (Table 4). For the 25 samples with a plasma VL ranging from >5000 to 10,000 copies/mL, 22 (88%) were detected by the DBS sample. For the 200 samples with a plasma VL > 10,000 copies/mL, 196 (98%) were detected by the DBS sample.

**Table 3:** Misclassification of Abbott DBS viral load compared with paired Roche plasma VL among children on ART in Mozambique.

Roche Plasma viral load (copies/mL)				Abbott DBS VL (copies/mL)				
	Total samples	<1,000	1,000–5,000	>5,000–10,000	>10,000–100,000	>100,000	Detection Rate at 1000 copies/mL (%)	95% CI
<1,000	420	411	7	1	1	0	2.1	(0.9, 4.9)
1,000–5,000	72	52	18	1	1	0	27.8	(13.4, 48.9)
>5,000–10,000	25	3	17	3	2	0	88	(46.9, 98.4)
>10,000–100,000	150	3	14	34	99	0	98	(94.4, 99.3)
>100,000	50	1	0	1	22	26	98	(75.1, 99.9)
Total samples	717	470	56	40	125	26		

**Table 4:** Plasma viral load ranges of the 72 DBS samples with plasma VL ranging from 1000 to 5000 copies/mL.

Paired Plasma VL	# of detectable DBS using 1000 copies/mL as threshold	# of undetectable DBS using 1000 copies/mL as threshold
1000	39	38
1001–2000	7	3
2001–3000	10	3
3001–4000	8	6
4001–5000	8	2
Total	72	52

## Discussion

In order to reach the ambitious 90-90-90 global goals, with 90 percent of people with HIV diagnosed, 90 percent on ART, and 90 percent virally suppressed by 2020 (UNAIDS, 2013), more people with HIV in RLS will need to initiate and stay on ART, and more of them will need active ART management and access to detection of treatment failure. Because conventional VL testing using plasma is often unavailable or impractical in RLS, use of a DBS sample is a feasible and practical alternative. Therefore, it is important to understand the potential limitations of using DBS for VL testing, especially for the WHO recommended treatment failure threshold. In this study, the performance of one FS-DBS spot to determine HIV treatment failure was evaluated using paired DBS and plasma samples from children on ART in Mozambique.

Our data showed that one DBS VL spot performed relatively well, with specificities of 97.8%, 99.0%, 99.5%, and low false positive rates (2.2%, 1.0%, and 0.5%) rates at all three thresholds (1000, 3000, 5000 copies/mL) (Table 2). We found lower sensitivities, higher specificities and lower false positive rates than those reported by Schmitz et al (94.5%, 97.8%, 98.8%, and 6.3, 2.7, 1.4, respectively) [20]. The differences in the specificities can be explained by sample input. In Schmitz’s study two DBS spots were used per test [20], whereas, in our study, one DBS spot was used. When sample input increases, the test sensitivity may increase but the specificity often decreases as a trade-off. Based on our data, we anticipate that few children not failing treatment will be misclassified as failing treatment using one DBS sample.

The sensitivity of one DBS VL spot (79.9%) was highest when 1,000 copies/ml was used as the threshold (Table 2). The DBS VL misclassification rate below this threshold was very low. Among the 419 plasma samples below the threshold, only 9 were misclassified by one DBS spot (Table 3). Among the 225 children with virologic failure, 96.9% (218/225) were detected. However, one DBS spot led to a higher misclassification rate when the plasma VL was between 1000 and 5000 copies/mL. In this range, 72.2% (52/72) of treatment failures were undetected (Table 3). Among these 52 DBS spots that were undetectable, 38 (73.1%) had plasma VL at 1000 copies/mL (Table 4), exactly at the WHO-recommended threshold. Considering the sensitivities and specificities at all evaluated thresholds, 1000 copies/mL appears the better threshold choice for treatment monitoring and treatment failure determination using DBS since 1000 copies/mL had the highest sensitivity and the specificity was very good at all three thresholds. However, we caution that since the sensitivity of one DBS spot was not perfect, 20 out of 100 children with a viral load >1000 may be misclassified as having an undetectable viral load (false negative). Although this group of children on ART may not be representative of the viral load distribution in other populations, the low detection rate or higher misclassification rate using one DBS spot for plasma VL between 1000 and 5000 copies/mL presents a challenge for clinicians if a single DBS spot for VL test is used for patient monitoring. This would be of concern in settings with little clinical follow-up or adherence counseling. WHO recommends continued ART monitoring at 6, 12 months after ART initiation followed by annual VL testing [26]. WHO and some studies also recommend intensive adherence counseling to optimize treatment adherence and reduce the risk of treatment failure [27–30]. An additional alternative is to use two DBS samples as in Schmitz’s study [20] instead of only the one that we used. At the thresholds of 1000, 3000, and 5000 copies/mL, Schmitz’s study reported higher sensitivities using two FS-DBS (88.1%, 85.2% and 82.2%), respectively [20]. The finding that many of the false negatives (52 of the 72) had viral loads between 1000 and 5000 copies/ml suggests that close follow up and repeat testing may be needed, because viral loads in this range may indicate poor adherence and increased risk for developing low CD4 counts and antiretroviral resistance [31, 32].

The Kappa value of 0.8 (0.73, 0.87) at 1000 copies/mL was higher than at 3000 or 5000, confirming the superiority of 1000 as the optimal DBS threshold (Table 2). The mean difference between the Roche plasma and Abbott DBS VL results (0.311 in Log _10_) in Fig 1 is comparable to the mean difference we have observed between Roche and Abbott plasma VL results [33] and the mean difference in the Roche and Abbott package inserts [21, 24]. The range of the 95% confidence limits was between -0.908, and 1.530, mainly because 67 of 717 samples showed greater VL discrepancies between the plasma and DBS. However, most of these samples (48/67) had a plasma VL below 1000 copies/mL.

One limitation of our study was that, because of local resources, the paired plasma and DBS samples could not be processed with the same VL test on the same instrument; the plasma samples were processed with Roche’s plasma VL test and the DBS samples were processed with Abbott’s DBS VL test. Since the plasma VL results were used as the reference standard in the evaluation study, the variability between the Roche and Abbott plasma VL results may complicate our evaluation. However, several studies have indicated that the Roche and the Abbott HIV VL testing methods are comparable [33–36]. In particular Nguyen et al. [33] published international HIV viral load (VL) proficiency testing (PT) program data from 2010 to 2012using dried tube specimens (DTS) with spiked HIV-1 virus. The PT program was conducted 2–3 times a year and up to 114 laboratories in 44 countries participated by the end of 2012. The concentration of 5 samples in each PT panel ranged from 10^2^ to 10^6^ (Log_10_ value). Between 2010 and 2012, 454 and 770 samples “passed” PT criteria using the Abbott and Roche HIV-1 plasma VL testing methods, respectively. The VL results from the two methods showed a correlation coefficient (*R*^*2*^) value of 0.97, indicating excellent agreement [33]. Based on these data, we believe that Roche plasma VL results can be used as the reference standard to evaluate the performance of a DBS VL test using the Abbott platform.

In order to ensure the quantity and quality of the DBS samples, the FS blood was collected using a Micro-EDTA tube and dotted on filter paper using a plastic transfer pipette. The advantage of this method is that it provides enough time to collect a good quantity of blood from the figure-stick to dot the DBS card without concern about blood coagulation. The VL result variation between the venous blood and the FS blood was discussed in a DBS VL evaluation study conducted in Kenya[20]. Data indicated that there was no significant difference in the VL results between the venous DBS and the FS-DBS.

Recently, Abbott Molecular received a Conformité Européene (CE) mark for their DBS VL test by adding DBS as another sample type. The new Abbott DBS VL test uses one DBS spot with 70–75 ul of blood per test. The extraction procedure in the Abbott CE mark DBS VL differs from the DBS VL methods we used in this study [22]. The new Abbott CE mark DBS VL reports a limit of detection of 839 copies/mL, a sensitivity of 94.1%, and specificity of 96.3% [22]; at 1000 copies/mL, it shows higher sensitivity (94.1%) but slightly lower specificity (96.3%) than what we found (79.9%, 97.8%) in this study. Several other VL tests using DBS, including bioMerieux NucliSENS EasyQ HIV v2.0 using 2 spots of 50 μL of blood [37], VERSANT HIV-1 RNA 1.0 (kPCR) using 1 spot of 50 μL [38], and Roche COBAS Ampliprep/COBAS TaqMan HIV-1 Test 2.0 (free virus elution protocol) using 1 spot of 70 μL [39], have also passed the World Health Organization (WHO) prequalification *In vitro* Diagnostics Devices (IVD) process, multi-country evaluation studies, and are recommended for HIV VL testing. When a country or program, based on local resources, selects a commercially available DBS VL test, performance data, particularly VL test performance data from local patients receiving ART, should be carefully considered. If a country or program selects a WHO-recommended VL test, a small method validation is recommended prior to use.

In conclusion, because of the sensitivity (80%) and specificity (98%) of VL testing using one FS-DBS with the Abbott VL test, this method may be considered as part of a VL monitoring program and 1000 copies/mL may be used to determine treatment failure among ART‐experienced children in RLS where the ability to process plasma samples is not available. However, evaluation of this method using two samples may show better sensitivity than what we found, and a careful treatment monitoring program should be in place to closely monitor all children with DBS VL results below 1000 copies/mL.

## Disclaimer

This research has been supported by the President’s Emergency Plan for AIDS Relief (PEPFAR) through the Centers for Disease Control and Prevention. This information is distributed solely for the purpose of predissemination peer review under applicable information quality guidelines. It has not been formally disseminated by the Centers for Disease Control and Prevention/Agency for Toxic Substances and Disease Registry, Atlanta, Georgia, USA. Instituto Nacional de Saúde (INS), Maputo Central Hospital, Ministry of Health Fundação Ariel Glaser contra o SIDA Pediátrico (Ariel). The findings and conclusions in this report are those of the authors and do not necessarily represent the official position of the US Centers for Disease Control and Prevention. Use of trade names is for identification purposes only and does not constitute endorsement by the U.S. Centers for Disease Control.
